# An examination of periodontal treatment and per member per month (PMPM) medical costs in an insured population

**DOI:** 10.1186/1472-6963-6-103

**Published:** 2006-08-16

**Authors:** David A Albert, Donald Sadowsky, Panos Papapanou, Mary L Conicella, Angela Ward

**Affiliations:** 1 Columbia University College of Dental Medicine, New York, New York, USA; 2 Aetna, Fairfield, New Jersey, USA

## Abstract

**Background:**

Chronic medical conditions have been associated with periodontal disease. This study examined if periodontal treatment can contribute to changes in overall risk and medical expenditures for three chronic conditions [Diabetes Mellitus (DM), Coronary Artery Disease (CAD), and Cerebrovascular Disease (CVD)].

**Methods:**

116,306 enrollees participating in a preferred provider organization (PPO) insurance plan with continuous dental and medical coverage between January 1, 2001 and December 30, 2002, exhibiting one of three chronic conditions (DM, CAD, or CVD) were examined. This study was a population-based retrospective cohort study. Aggregate costs for medical services were used as a proxy for overall disease burden. The cost for medical care was measured in Per Member Per Month (PMPM) dollars by aggregating all medical expenditures by diagnoses that corresponded to the International Classification of Diseases, 9^th ^Edition, (ICD-9) codebook. To control for differences in the overall disease burden of each group, a previously calculated retrospective risk score utilizing Symmetry Health Data Systems, Inc. Episode Risk Groups™ (ERGs) were utilized for DM, CAD or CVD diagnosis groups within distinct dental services groups including; periodontal treatment (periodontitis or gingivitis), dental maintenance services (DMS), other dental services, or to a no dental services group. The differences between group means were tested for statistical significance using log-transformed values of the individual total paid amounts.

**Results:**

The DM, CAD and CVD condition groups who received periodontitis treatment incurred significantly higher PMPM medical costs than enrollees who received gingivitis treatment, DMS, other dental services, or no dental services (p < .001). DM, CAD, and CVD condition groups who received periodontitis treatment had significantly lower retrospective risk scores (ERGs) than enrollees who received gingivitis treatment, DMS, other dental services, or no dental services (p < .001).

**Conclusion:**

This two-year retrospective examination of a large insurance company database revealed a possible association between periodontal treatment and PMPM medical costs. The findings suggest that periodontitis treatment (a proxy for the presence of periodontitis) has an impact on the PMPM medical costs for the three chronic conditions (DM, CAD, and CVD). Additional studies are indicated to examine if this relationship is maintained after adjusting for confounding factors such as smoking and SES.

## Background

Systemic health is often associated with the condition of the oral cavity, because many systemic diseases manifest in the mouth. However, less is known about the connection between a diseased periodontium and the impact it may have on systemic health. The concept that periodontal disease is a localized condition that affects only the teeth and the surrounding tissue and bone is being increasingly questioned and examined. The connection between a diseased periodontium and its impact on systemic health is currently being investigated for many conditions including cardiovascular diseases and diabetes. About 50 percent of the adults in the United States have gingivitis (gum inflammation), 35 percent have some form of periodontitis, and 7 to 15 percent have severe periodontal disease (inflammation of the gums leading to destruction of the bone supporting the teeth) [1,2] consequently, the association between periodontal infection and systemic health has important implications for the treatment and management of patients.

Cardiovascular disease is a leading cause of death within the United States and accounts for 40 percent of all deaths. Over 927,000 Americans die of cardiovascular disease each year, while over 70 million (over one-fourth of the population) live with the condition. Coronary heart disease is a leading cause of premature, permanent disability in the United States workforce. Health care expenditures and lost productivity from death and disability due to cardiovascular disease is projected to be $394 billion in 2005 [3].

A number of epidemiological studies have examined the relationship between periodontal disease and vascular disease [4-8]. Beck et al. analyzed data from a cohort study and reported that the incidence odds ratios for alveolar bone loss associated with severe periodontal disease and total coronary heart disease, fatal coronary heart disease and stroke were 1.5, 1.9, and 2.8 respectively. The researchers concluded that an increased level of bone loss was accompanied by a higher occurrence of heart disease, which suggested an association between the two conditions [4]. Genco et al. reported similar findings. In this study, a group of Native Americans from the Gila River Indian Community had their alveolar bone level and cardiovascular status monitored prospectively over a period of ten years. Among all age groups, the researchers found that bone level was predictive of cardiovascular disease, with an odds ratio of 2.68 in groups ≤ 60 years [6]. A cross-sectional study conducted by Arbes et al., analyzed data obtained from the National Health and Nutrition Examination Survey (NHANES III) to determine whether an association between periodontal disease and coronary heart disease could be found. Results from this study supported a periodontal disease/coronary heart disease connection [8]. In two separate case-control studies Mattila et al. compared patients with acute myocardial infarction with a control group that was selected from the community at random. The results from both studies indicated that patients with acute myocardial infarction were found to have poor oral conditions, while those in the control group had better oral health [7]. The connection between periodontal disease and cardiovascular disease appears to be further supported by the findings from a study conducted by DeStefano et al. This study, which followed its subjects for fourteen years, included an examination of several variables that included age, gender, systolic blood pressure, total cholesterol levels, physical activity and periodontal disease. Of the 10,000 subjects analyzed, the researchers found that controlling for other variables those with severe periodontal disease had a 25 percent increased risk of developing coronary heart disease than those who had only mild periodontitis [5]. Wu et al., examined the relationship between periodontal health and its association with nonfatal or fatal cerebrovascular accidents (CVA). The results of this cohort study, in which data were obtained from the First National Health and Nutrition Examination Survey (NHANES I) and from its follow-up study, showed periodontitis to be a significant risk factor for total CVA, particularly non-hemorrhagic stroke [9]. Desvarieux et al found a direct relationship between specific periodontal microbiota and subclinical atherosclerosis [10].

In 2003 the United States Department of Health and Human Services, Centers for Disease Control and Prevention, reported that 18.2 million people in the United States have diabetes mellitus and that each year an additional one million adults are diagnosed with the condition [11]. Diabetes is the sixth leading underlying cause of death in the United States and has been estimated to cost 91.5 billion dollars annually in medical care and lost productivity [12]. The prevalence of diabetes has increased 30 to 40 percent during the past two decades [13] and it is expected that the number is likely to become larger as the population grows older and obesity becomes an increasing problem.

Evidence suggests that diabetes is associated with the increased occurrence and progression of oral complications that include gingivitis, periodontitis, periapical abscesses, and alveolar bone loss. Among the many oral problems that can occur because of diabetes, the prevalence of severe periodontitis is significantly higher among people with poorly controlled diabetes [14] to the extent that periodontitis has been called the "sixth complication of diabetes" [15]. The reason for the higher rates of periodontal disease in people with diabetes is not completely understood, but studies have reported that there is little difference in the periodontal flora of people with and without diabetes, and suggest that the increased destruction of tissue among those with diabetes may be due to an altered host susceptibility to periodontal pathogens mediated by the accumulation of Advanced Glycation End Products in the tissues, as well as microvascular changes and perhaps impaired lipid metabolism [16-18]. Conversely, these data also suggest that the presence of periodontal infection can adversely affect glycemic control in people with diabetes and that there appears to be a bi-directional relationship between the two conditions. The exact nature of this relationship is not yet clear, but there is evidence indicating that the management and treatment of periodontal disease in patients with poorly controlled diabetes may reduce their insulin requirements, and improve glycemic control and overall metabolic balance. Grossi et al. reported that adults with diabetes who received dental cleanings in combination with systemically administered antibiotics showed a significant improvement in their condition and a reduction in their glycated hemoglobin [19]. The findings from a study conducted by Taylor et al., also appears to support the concept that the presence of periodontal disease can have an adverse impact on glycemic levels in people who have diabetes. These data indicate that people with non-insulin-dependent diabetes mellitus and severe periodontitis had an increased risk of developing poor glycemic control [20]. Stewart suggested that periodontal therapy was associated with improved glycemic control in persons with type 2 diabetes [21]. Rodrigues demonstrated that non-surgical periodontal therapy improved glycemic control in patients with type 2 diabetes [22]. A review of the literature that examined twelve studies conducted to determine the effects of treating periodontal diseases on glycemic control, concluded that the presence of periodontal disease can lead to poor glycemic control in patients with diabetes and that treating their periodontal disease could have a beneficial impact on glycemic control in those with type 1 or 2 diabetes [23].

In this study, dental and medical claims data from the Aetna Integrated Informatics Data Warehouse were examined to determine if there were differences in total medical costs for cardiovascular diseases [Coronary Artery Disease (CAD), and Cerebrovascular Disease (CVD)], and diabetes mellitus (DM) condition members receiving the following dental services: (1) periodontal treatment (periodontitis or gingivitis); (2) regular dental maintenance services (DMS); (3) other dental services; or (4) no dental services. Claims for specific medical diagnoses groups were extracted from the medical claims database and dental procedure claims were taken from the dental claims database.

## Methods

The source of data for this report is Aetna's Data Warehouse for all preferred provider organization insurance members with dates of service from January 1, 2001 to December 31, 2002. Members who were included in the sample were selected if they had concomitant and continuous medical and dental coverage in an Aetna Dental and Medical product. Demographic information available with this database included age and gender. Race/ethnicity was not available for this dataset. In 2002, it was reported in the United States that 71% of Whites, 42% of Latinos, 50% of African Americans, 61% of Asians and 41% of American Indian/Alaskan Natives had employer-sponsored health insurance [24]. Overall, females' made up 52% of the total population. The mean age for members with DM was 57.2 years, with CAD was 63.5 years, and CVD was 68.5 years. The sample reflected overall Aetna membership within the United States with 17% from the Mid-Atlantic, 16% from the Northeast, 15% from the North Central region, 14% from the Southeast, 12% from the Southwest, and 12% from the West. 14% were not assigned to a region of the United States. Over half (58%) of the medical membership at Aetna participates in a preferred provider organization (PPO) product (8,360,093). General dentists (44,762) and periodontists (6,491) participate in the Aetna PPO.

The sample was derived from a larger pool that included 1,171,122 unique medical members, and 1,105,030 unique dental members. 764,684 members with two years of continuous dental and medical coverage were potentially available. From this group 116,306 members qualified for inclusion in the study. These members had at least one of three chronic conditions: (1) diabetes mellitus (DM); (2) coronary artery diseases (CAD); and (3) cerebrovascular diseases (CVD). These three groups were not mutually exclusive (Table 1). The International Classification of Diseases, 9^th ^Edition (ICD-9) codebook was used to define these conditions (Table 2) [25].

To evaluate the potential relationship between periodontal disease treatment and chronic conditions all PPO members were placed into one of the following mutually exclusive categories, based on their utilization of dental services: (1) members who had treatment for periodontal disease, (periodontitis treatment or gingivitis treatment) (2) members who had at least one dental maintenance service (examination and preventative treatment), but no periodontal treatment (DMS); (3) members with other dental services (restorative, prosthetic, and surgical treatment), but no dental maintenance services and no periodontal services; and (4) members with no dental services at all. The total medical per member per month (PMPM) medical cost for each of these categories was calculated. The American Dental Association's CDT-3 codebook was used to define these treatment categories (Table 3) [26]. The dental codes listed in the CDT-3 are treatment codes. The CDT-3 codebook does not record disease status. Codes from 4000–4999 encompass all periodontal treatment procedures (Table 3). The periodontal treatment category was then dichotomized into periodontitis or gingivitis treatment. If no periodontitis treatment was provided, the enrollees in the periodontal treatment group were assigned to the gingivitis group.

### Statistical analysis

To control for differences in the overall disease burden of each group, a previously calculated retrospective risk score utilizing Symmetry Health Data Systems, Inc. Episode Risk Groups™ (ERGs) was obtained from the insurers' data warehouse for each selected member for each year of the study, and the average of these risk scores was calculated for each group [27-29]. ERGs use basic inputs such as the diagnoses recorded on medical claims and demographic variables to predict health risk. A key feature of ERGs is its use of episodes of care as markers of total health burden risk rather than the diagnoses from individual medical encounters. By using episodes of care, the focus is placed on the key information describing a patient's underlying medical condition rather than the individual services provided in its treatment [27]. The individual average risk scores were used to determine the relative risk of each group compared to the population average, and then they were used to adjust the actual PMPM medical cost at the member level. Using the average of these adjusted scores permitted the groups to be compared with disease burden "adjusted out". The score is calculated for each member in each year based on the member's age, gender, and diagnosed conditions. The score is proportional to the expected annual medical cost. For example, if Member A has a score that is 10% higher than Member B, then Member A would be expected to have annual medical claim costs that are 10% higher than Member B, all else being equal. Approximately 7% of the sample was dropped because one or both of the retrospective risk scores was missing.

The differences between group means were tested for statistical significance using log-transformed values of the individual total paid amounts. The two-sample t-test assumes that the values being compared are normally distributed, and experience has shown that total paid amounts for individuals are not normally distributed. This is typical of medical costs, where medical costs increase at a higher rate among those with greater illness or more severe chronic conditions. When total paid amounts are logged the result is reasonably close to a normal distribution, allowing the use of the t-test.

For each pair of groups, the first test performed determined whether the variances of the groups were significantly different. If they were, the Satterthwaite test was used to compare the means. If the variances were not significantly different, the two-sample t-test was performed. The comparisons used two-tailed tests.

The Columbia University Institutional Review Board reviewed the study protocol and determined it to be exempt from review, because there was no interaction with subjects, no intervention, and no private, identifiable information was collected.

## Results

Periodontal treatment was provided to 14% of members with a DM diagnosis, 13.12% with a CAD diagnosis, and 11.5% with a CVD diagnosis. Less than 10% of the periodontal patients were treated for periodontitis (as defined in Table 1), during the two-year period that was examined by this study. Approximately half of the patients within the three chronic conditions had DMS procedures. Nearly 30% of each chronic condition group had no dental services.

Overall 6.7% of the members were diagnosed with DM, 9.2% with CAD, and 2.9% with CVD. The mean age for members with DM was 57.2 years, with CAD was 63.5 years, and CVD was 68.5 years. Of the female members 35.4% were diagnosed with DM, 33% with CAD, and 42.6% with CVD. The age and percent female for each of the chronic conditions exhibited no significant variation within the periodontal treatment categories, the DMS category, or the no dental services category.

PMPM medical expenditures included all ICD-9 codes that were billed for a member within a chronic condition category (Table 4). The PMPM medical costs were paid as per plan design of coverage. Table 4 displays for each group, the average PMPM medical cost for all medical (but not dental) services incurred during the two-year period. PMPM medical expenditures for the DM, CVD, and CAD groups with periodontitis treatment exhibited significantly higher log adjusted PMPM medical costs than for groups with gingivitis, DMS, other dental services or no dental services (p < .001). The DM gingivitis treatment group exhibited significantly higher log adjusted PMPM medical costs than for groups with other dental services (p = .033), or no dental services (p < .001). For the cardiovascular conditions groups with gingivitis treatment, a marginally significant difference in PMPM medical costs was found only for the CAD gingivitis group compared to the other dental services group (p = .048). All periodontitis treatment groups had significantly higher PMPM medical costs when compared to gingivitis treatment groups (p < .001).

The mean PMPM medical costs were skewed to the right (towards higher costs). To normalize the data a log transformation was conducted. After log transformation of the data and application of Episode Risk Groups™ (ERG), average retrospective scores were obtained for each of the three chronic conditions (Table 5). The retrospective risk for DM, CAD, and CVD groups with periodontitis disease treatment were significantly lower (p < .001), when compared to groups who had only DMS procedures, other dental services or no dental services. A lower retrospective risk was observed for DM, CAD, and CVD groups who had periodontitis treatment than for those who had gingivitis treatment only (p < .001).

In Table 6, the untransformed PMPM medical cost for individuals are risk-adjusted using the average risk score for each group, and these are log-transformed for the t-test. The risk scores from Table 5 have been applied to the PMPM medical expenses from Table 4 to produce risk-adjusted PMPM medical costs. When the PMPM medical costs were adjusted for risk, the costs for groups who received periodontitis disease treatment were higher for all three chronic conditions, when compared to members who had only DMS procedures, other dental services or no dental services (p < .001). DM, CAD, and CVD periodontitis treatment groups had significantly higher expenses than gingivitis treatment groups (p < .001). The DM, CAD, and CVD risk adjusted gingivitis treatment groups had significantly higher PMPM medical costs than enrollees with other dental services, or no dental services.

## Discussion

DM, cardiovascular diseases (CAD and CVD) and periodontal disease are common in the population. The observed associations between the diseases do not imply that periodontal disease has a causal association with DM or the cardiovascular diseases. Periodontitis, DM and cardiovascular diseases share many risk factors, including age, smoking, and SES. Any relationship observed may be the result of confounding biases. The age of enrollees was similar, however smoking, SES and other factors were not available in this data set.

Although we know that the periodontitis treatment groups did have periodontal disease, we do not know how long they had the disease before their treatment or the severity of their disease without sequential clinical examinations. Since periodontal disease is a chronic condition whose prevalence increases with age it is reasonable to conclude that significant chronic periodontal disease burden existed for the three chronic disease condition members (DM, CAD, and CVD). Because CDT-3 coding is a treatment system and does not list conditions we are not able to assess overall dental disease burden or severity in this retrospective study. It should be emphasized that minimal demographic and social information (age and sex only) was available for this population. Additional investigation is warranted to examine periodontal health indicators such as bleeding, and pocket depth.

Higher log transformed medical costs for the DM group with periodontitis treatment were observed during the two-year study period in comparison to all other dental service groups or to the group with no dental services. The DM periodontitis treatment group also exhibited significantly higher PMPM medical costs when compared to the gingivitis treatment group. Patients who receive periodontitis treatment would be expected to exhibit more bone loss and inflammation compared to patients with gingivitis [19-23]. Without intraoral periodontal examinations and the examination of confounders such as smoking it is difficult to explain the reason for the increase in medical costs in groups with periodontitis. A contributory factor may be diminished glycemic control in patients in the DM periodontitis treatment group when compared to the other dental treatment groups.

The log transformed PMPM medical costs for the CAD and CVD groups were higher for the groups with periodontitis treatment than for all other dental service groups or to the group with no dental services. Periodontitis has been associated with thickening of arterial walls. Desvarieux and colleagues observed an increase in carotid intima thickening in patients diagnosed with periodontitis [10]. Periodontitis is a chronic disease that may affect the cardiovascular system either through bacteria associated with periodontal disease entering the circulation and directly contributing to the accumulation of lipid containing plaques on the inner most layer of the artery wall; or to systemic factors that can alter the immunoinflammatory process [4,5]. Hujoel et al., in an examination of the first National Health and Nutrition Examination Survey (NHANES) found that gingivitis was not associated with CAD, while periodontitis was weakly associated [30]. For gingivitis (no periodontitis treatment) groups no association was found for unadjusted PMPM medical expenditures (Table 4) in the CAD and CVD conditions compared to the no dental services group, however the risk adjusted gingivitis groups (Table 6) were associated with higher costs.

Episode Risk Groups™ (ERGs) utilize "episodes of care" as a marker of risk rather than the ICD-9 codes from the medical encounter. The ERG provides a risk assessment of the expected health care costs or utilization of a group of individuals [29]. For DM and the cardiovascular disease groups (CAD and CVD), PMPM medical costs were higher in the periodontitis treatment group than the groups with no dental services (p < .001). However, the calculated ERG scores for the DM and cardiovascular disease groups were significantly different in the opposite direction, with less risk assigned to the periodontitis treatment group than to the no dental services group (p < .001). It is possible that dentist discretion to treat or not treat existing periodontal conditions was a confounding factor. Individual dentist's knowledge of systemic conditions could influence treatment plans, recall patterns and the decision to treat periodontal disease. It is plausible that individuals in the periodontitis treatment groups differed from patients who did not receive periodontal treatment on behavioral factors such as health seeking behaviors. The predictive ability of the ERG model can be limited for patients with higher costs or chronic medical conditions such as those described in this study [28]^.^

It should be noted that both the unadjusted (Table 4) and risk adjusted (Table 6) PMPM medical costs for the periodontitis treatment groups were significantly higher than the dental services treatment groups or no dental services group for all three conditions (DM, CAD and CVD) (p < .001). The unadjusted PMPM medical costs for the gingivitis group compared to other dental services or no dental services was significant for the DM condition only. After risk adjusted all chronic condition gingivitis groups (DM, CAD, and CVD) had significantly higher PMPM medical costs than other dental services or no dental services groups.

The need for treatment of periodontal disease in the general population is underscored by the increasing prevalence of periodontal disease with age. The prevalence of periodontal disease in the population from age 15–24 years is approximately 30%; this increases to over 70% in the population from age 45–54 years. It is estimated that at least 35% of the dentate U.S. adults aged 30 to 90 have periodontitis, with 21.8% having a mild form and 12.6% having a moderate or severe form [1,2].

There are a number of additional limitations that should be considered when interpreting the findings from this study. First, the data presented here are from members of a large insurance carrier who had continuous medical and dental PPO coverage for a two-year period (764,288 unique members), and they therefore may not represent the overall insured population for this company. Although all patients were PPO members there is some variability within plans. This may have accounted for some of the observed differences. Members who did not maintain continuous coverage over the two-year study period were not included in the analysis. Second, women are underrepresented in this sample in all three chronic conditions, and do not reflect the fairly equal distribution of these diseases in the overall population. For example, in the general population for heart disease the crude percent for all types of disease are 11.4% for males and 10.7% for females, for stroke 2.4% for males and 2.3% for females, and for diabetes 7.0% for males and 6.2% for females [31]. Third, the utilization of dental services by the study sample was higher than the general population. For example, 70.6% of the members with diagnosed diabetes saw a dentist, compared to 65.8% of adults reported within 1995–1998 Behavioral Risk Factor Surveillance System [32]. Caution should be used in generalizing findings presented here to the insured population or to the general U.S. population.

## Conclusion

In this retrospective examination of patient data we found periodontitis treatment groups had a lower retrospective risk for their chronic condition (DM, CAD, or CVD), than patients who did not have periodontitis treatment. There is evidence that an individual's systemic condition can affect their overall oral health, and a mounting body of evidence that oral health, particularly periodontal status can also affect an individual's general health status. It may be reasonable, therefore, to recommend that examination of the oral cavity be included in guidelines for care of patients with DM, CAD and CVD, and that public health programs and insurers work together to raise awareness of the need for periodic dental visits for those members of the population who have diabetes and cardiovascular diseases. This was recognized in objective 5.15 of Healthy People 2010, which calls for increasing the proportion of people with diabetes who have at least an annual dental examination [33].

## Competing interests

Mary Conicella is an employee of Aetna. The researchers and authors at Columbia University (David Albert, Donald Sadowsky, Panos Papapanou, and Angela Ward) do not receive remuneration from Aetna. The research was supported by a grant from Aetna.

## Authors' contributions

MC and DA carried out the project. DA, PP, MC, and DS worked on the project plan, and interpretation of results. AW contributed to the background section and reviewed and edited the manuscript. All authors helped to draft the manuscript and have approved the final manuscript.

## Pre-publication history

The pre-publication history for this paper can be accessed here:



## Figures and Tables

**Table 1:** Number of members with specified conditions grouped by utilization of dental services (n = 116,306)^a^

**Chronic Condition**^b^	**Periodontal Treatment**^c^	**Periodontitis Codes**	**Gingivitis Codes**	**Dental Maintenance Services (DMS)**^d^	**Other Dental Services**^d^	**No Dental Services**^d^
**Diabetes (n = 51,560)**	7,232	3,829 (7.4%)	3,403 (6.6%)	27,699 (53.7%)	1,473 (2.9%)	15,156 (29.4%)
**Coronary Artery Disease (n = 75,262)**	9,882	4,783 (6.4%)	5,099 (6.8%)	42,990 (57.1%)	2,341 (3.1%)	20,049 (26.6%)
**Cerebrovascular Disease (n = 22,153)**	2,564	1,109 (5.0%)	1,455 (6.5%)	12,367 (55.8%)	801 (3.6%)	6,421 (29.0%)

^a^Data are cumulative for two-year period (2001 and 2002)

^b^Chronic Condition categories are not mutually exclusive

^c ^Periodontal treatment includes all D4000–4999 codes (periodontitis and gingivitis)

^d^Enrollees in the dental maintenance services, other dental services, or no dental services received no periodontal services in 2001 and 2002

**Table 2:** Chronic conditions and their associated ICD-9 diagnosis and procedure codes^a^

**Category**	**Code Type**	**ICD-9 codes**
Diabetes	*ICD-9 diagnosis*	250–250.93, 357.2, 362.0–362.02, 366.41
Coronary Artery Disease	*ICD-9 diagnosis*	402–402.91, 410–414.9, 428–428.9, 429.0–429.2, 429.7–429.79, 440.0–440.9, 443.0–443.9, 444.0–444.9
Cerebrovascular Disease	*ICD-9 diagnosis*	433–437.19, 437.4–437.49, 437.6–437.69, 437.9–438.9

^a ^International Classification of Diseases, Ninth Revision, Clinical Modification (ICD-9-CM), Sixth Edition, U.S. Department of Health and Human Services. Centers for Disease Control and Prevention, National Center for Health Statistics. Hyattsville, MD

**Table 3:** Dental utilization classification and their associated CDT-3 treatment codes^a^

**Category**	**Code Type**	**CDT-3 codes**
Periodontal Treatment^b^	*CDT-3*	D4000 – D4999
Periodontitis Codes	*CDT-3*	D4220, D4240, D4245, D4260, D4263, D4264, D4266, D4267, D4268, D4274, D4341, D4381
Gingivitis Codes	*CDT-3*	All other D4000–D4999 codes
Dental Maintenance Services (DMS)^c^	*CDT-3*	D0120, D0150, D0180, D0210–0470, D1110–1205, D1330, D0140, D0160, D0170, D0472, D1310–1320, D1351
All Other Dental Services^d^	*CDT-3*	D5000–9974, D0473–0999, D1352–3999

^a ^CDT-3: Current Dental Terminology: Users Manual/American Dental Association. Version 2000, Chicago, Ill.: American Dental Association, 1999

^b^Includes all codes related to periodontal treatment including scaling and root planing and periodontal surgical procedures

^c^Includes examination and preventative treatment

^d^Includes restorative and prosthetic treatment

**Table 4:** Total medical per member per month costs with specified conditions grouped by utilization of dental services^a^. Total medical per member per month costs with specified conditions grouped by utilization of dental services^a,c^

		**Diabetes**	**Coronary Artery Disease**	**Cerebrovascular Disease**
**Periodontitis Codes**		$342.48 ($4.71)	$481.64 ($4.97)	$579.82 ($4.98)
**Gingivitis Codes**		$361.95 ($4.58)	$418.34 ($4.62)	$475.62 ($4.53)
**Dental Maintenance Services (DMS)**^b^		$347.89 ($4.56)	$416.64 ($4.58)	$418.38 ($4.48)
**Other Dental Services**^b^		$439.02 ($4.45)	$489.89 ($4.51)	$405.24 ($4.39)
**No Dental Services**^b^		$387.93 ($4.44)	$469.19 ($4.59)	$485.35 ($4.54)

**P-values for Periodontitis Codes**	Periodontitis Codes vs. DMS	**< .001**	**< .001**	**< .001**
	Periodontitis Codes vs. Other Dental Services	**< .001**	**< .001**	**< .001**
	Periodontitis Codes vs. No Dental Services	**< .001**	**< .001**	**< .001**
**P-values for Gingivitis Codes**	Gingivitis Codes vs. DMS	0.596	0.216	0.379
	Gingivitis Codes vs. Other Dental Services	**0.033**	**0.048**	0.157
	Gingivitis Codes vs. No Dental Services	**< .001**	0.486	0.716
**P-values for Periodontitis Codes vs. Gingivitis Codes**	Periodontitis Codes vs. Gingivitis Codes	**< .001**	**< .001**	**< .001**

^a^Data are cumulative for two-year period (2001 and 2002)

^b^Enrollees in the dental maintenance services, other dental services, or no dental services received no periodontal services in 2001 and 2002

^c^The log-transformed values are shown in parenthesis. The differences between group means were tested for statistical significance using the log transformed values of the individual total paid amounts

**Table 5:** Episode Risk Group™ (ERG) scores for members with specified conditions grouped bu utilization of dental services ^a,c^

		**Diabetes**	**Coronary Artery Disease**	**Cerebrovascular Disease**
**Periodontitis Codes**		3.39	4.68	6.23
**Gingivitis Codes**		4.23	5.41	6.96
**Dental Maintenance Services (DMS)**^b^		4.18	5.51	7.06
**Other Dental Services**^b^		5.41	6.67	8.29
**No Dental Services**^b^		4.79	6.49	8.26

**P-values for Periodontitis Codes**	Periodontitis Codes vs. DMS	**< .001**	**< .001**	**< .001**
	Periodontitis Codes vs. Other Dental Services	**< .001**	**< .001**	**< .001**
	Periodontitis Codes vs. No Dental Services	**< .001**	**< .001**	**< .001**
**P-values for Gingivitis Codes**	Gingivitis Codes vs. DMS	0.580	0.170	0.560
	Gingivitis Codes vs. Other Dental Services	**< .001**	**< .001**	**< .001**
	Gingivitis Codes vs. No Dental Services	**< .001**	**< .001**	**< .001**
**P-values for Periodontitis Codes vs. Gingivitis Codes**	Periodontitis Codes vs. Gingivitis Codes	**< .001**	**< .001**	**0.002**

^a^Data are cumulative for two-year period (2001 and 2002)

^b^Enrollees in the dental maintenance services, other dental services, or no dental services received no periodontal services in 2001 and 2002.

^c^Retrospective risk score

**Table 6:** Risk-adjusted PMPM for members with specified conditions grouped by utilization of dental services^a, c^. Total medical per member per month costs with specified conditions grouped by utilization of dental services^a,c,d^

		**Diabetes**	**Coronary Artery Disease**	**Cerebrovascular Disease**
**Periodontitis Codes**		$158.67 ($3.95)	$161.63 ($3.89)	$146.28 ($3.62)
**Gingivitis Codes**		$ 134.57 ($3.60)	$121.57 ($3.41)	$107.36 ($3.07)
**Dental Maintenance Services (DMS)**^b^		$130.96 ($3.60)	$118.75 ($3.36)	$93.13 ($3.01)
**Other Dental Services**^b^		$127.47 ($3.23)	$115.52 ($3.10)	$76.80 ($2.78)
**No Dental Services**^b^		$ 127.26 ($3.35)	$113.71 ($3.22)	$92.40 ($2.94)

**P-values for Periodontitis Codes**	Periodontitis Codes vs. DMS	**< .001**	**< .001**	**< .001**
	Periodontitis Codes vs. Other Dental Services	**< .001**	**< .001**	**< .001**
	Periodontitis Codes vs. No Dental Services	**< .001**	**< .001**	**< .001**
**P-values for Gingivitis Codes**	Gingivitis Codes vs. DMS	0.954	0.057	0.250
	Gingivitis Codes vs. Other Dental Services	**< .001**	**< .001**	**< .001**
	Gingivitis Codes vs. No Dental Services	**< .001**	**< .001**	**0.017**
**P-values for Periodontitis Codes vs. Gingivitis Codes**	Periodontitis Codes vs. Gingivitis Codes	**< .001**	**< .001**	**< .001**

^a^Data are cumulative for two-year period (2001 and 2002)

^b^Enrollees in the dental maintenance services, other dental services, or no dental services received no periodontal services in 2001 and 2002.

^c^The untransformed PMPMs for individuals are risk-adjusted using the average ERG score, and these are log-transformed for the t-test. The log transformations immediately precede the t-tests.

^d^The log-transformed values are shown in parenthesis. The differences between group means were tested for statistical significance using the log transformed values  of the individual total paid amounts
